# Group cognitive remediation therapy for younger adolescents with anorexia nervosa: a feasibility study in a Japanese sample

**DOI:** 10.1186/s13104-017-2642-5

**Published:** 2017-07-25

**Authors:** Rie Kuge, Katie Lang, Ayano Yokota, Shoko Kodama, Yuriko Morino, Michiko Nakazato, Eiji Shimizu

**Affiliations:** 1 Support Center for Children and Family, Department of Psychology and Welfare, Tokyo Metropolitan Children’s Medical Center, 2-8-29 Musashidai, Fuchu-shi, Tokyo 183-8561 Japan; 2 Department of Cognitive Behavioral Physiology, Graduate School of Medicine Chiba University, 1-8-1 Inohana, Chuo-ku, Chiba-shi, Chiba 260-8670 Japan; 3 Psychological Medicine, Section Eating Disorders, Institute of Psychiatry, Psychology and Neuroscience, Kings’ College London, Basement, Ground and 1st Floor 103, Denmark Hill, London, SE5 8AZ UK; 4 Department of Clinical Psychology, Tokyo Metropolitan Matsuzawa Hospital, 2-1-1, Kamikitazawa, Setagaya-ku, Tokyo, 156-0057 Japan; 5 Department of Child and Adolescent Psychiatry, Tokyo Metropolitan Children’s Medical Center, 2-8-29 Musashidai, Fuchu-shi, Tokyo 183-8561 Japan; 6 Department of Psychiatry, Chiba University Graduate School of Medicine, 1-8-1 Inohana, Chuo-ku, Chiba-shi, Chiba 260-8670 Japan

**Keywords:** Adolescents, Anorexia nervosa, Cognitive remediation therapy, Group therapy, Neuropsychological assessments

## Abstract

**Objective:**

Cognitive remediation therapy (CRT) aims to increase patients’ cognitive flexibility by practicing new ways of thinking as well as facilitating bigger picture thinking, supporting patients with relevant tasks and encouraging an awareness of their own thinking styles. CRT has been applied in the treatment of adult anorexia nervosa (AN), and has been shown to be effective and acceptable. In adolescents, CRT has been piloted on both individual and group format. However, no studies are published in CRT for adolescents with AN in a Japanese sample. The objectives of this study were to assess the feasibility, to estimate effect sizes for the purpose of designing a larger study, and to assess the acceptability of a CRT group for younger adolescents with AN in a Japanese sample.

**Methods:**

Group CRT interventions were carried out with a total of seven adolescents with AN. Neuropsychological and psychological assessments (motivation, self-efficacy and depression) were administered before and after the group intervention. The participants completed worksheets (documents of participants’ thinking about their thinking style and the relation of the skills that they learnt through each session to real-life) and questionnaires after the group.

**Results:**

There were small effect sizes differences between the part of the pre and post neuropsychological tests and the pre and post ability to change (motivation). There were medium effect sizes differences between the pre and post depressive symptoms and importance to change (motivation). There was a large effect size shown between the pre and post weights. All participants were able to reflect on their own thinking styles, such as having difficulty with changing feelings and the tendency to focus on details in real-life. Adolescents’ feedback was positive, and the rate of dropout was low.

**Conclusion:**

CRT groups could be feasible and acceptable for younger adolescents with AN in a Japanese sample.

*Trial registration* UMIN No. 000020623. Registered 18 January 2016

## Background

Inefficiencies in set-shifting and central coherence are commonly observed in adult patients with anorexia nervosa (AN) [1, 2]. It is hypothesized that cognitive style may influence the maintenance of illness and response to treatment [2]. Although research on neuropsychological functioning of adolescents with AN is limited [3, 4], previous studies demonstrate that adolescent AN share the same cognitive processing inefficiencies as adult AN [5, 6].

Cognitive remediation therapy (CRT) aims to increase patients’ cognitive flexibility by practicing new ways of thinking as well as facilitating bigger picture thinking, supporting patients with relevant tasks and helping them to become aware of own thinking styles [7, 8]. CRT has since been applied for adult AN, showing to be effective and acceptable on both an individual basis and in group format [9, 10]. In adolescents, CRT has been piloted on both individual basis and in group format [9, 11–14]. The benefit of therapeutic interventions in a group context includes sharing and normalizing difficulties and generating a diversity of ideas and solutions from different perspectives [14]. However, no studies are published in CRT for adolescents with AN in a Japanese sample.

The objectives of this study were to assess the feasibility, to estimate effect sizes for the purpose of designing a larger study, and to assess the acceptability of a CRT group for younger adolescents with AN in a Japanese sample.

## Methods

### Study design and setting

This study adopted a single arm design. The study was conducted at the Department of Child and Adolescent Psychiatry Tokyo Metropolitan Children’s Medical Center (TMCMC), Tokyo, Japan.

### Participants

Seven participants took part in the study. Participants were recruited from inpatient wards at the Department of Children and Adolescent Psychiatry TMCMC. The Inclusion criteria were as follows: clinical diagnosis of AN according to Diagnostic and Statistical Manual of Mental Disorders, Fifth Edition criteria [15] by child psychiatrists aged 13–18 years, and hospitalized patients. All participants’ hospitalization was involuntary for medical care and protection. Exclusion criteria included Intelligence Quotient less than 70, history of brain injury, drug abuse/dependence, and critical physical disease. The participants were enrolled from January to April 2016. Two groups were run in total. The first and second group consisted of four and three adolescents respectively.

### Measures

Body mass index (BMI) and standard body weight ratio were assessed at admission, pre and post CRT group.

Neuropsychological measures were assessed pre and post CRT group.

#### The Rey–Osterrieth complex figure test (ROCFT) [16–18] (central coherence)

Participants were provided with a blank sheet of paper, a sheet with a complex figure and a pencil, and asked to copy the complex figure design. The drawing process was video-recorded. The ROCFT was scored according to Booth’s 2006 scoring method [19]. Order index (OI) yields a score between 0 and 3.33, with higher scores indicating drawing of global elements initially. Style index (SI) yields a score between 0 and 2, with higher scores indicating continuous drawing style for the main elements of the figure. OI and SI are computed and are added to give the central coherence index (CCI). CCI yields a score between 0 and 2, with higher scores indicating a more coherent drawing style. A second researcher who was independent of the study, co-rated 100% of the ROCFTs. Inter-rater reliability was almost perfect agreement (kappa = 0.91).

#### The Brixton spatial anticipation test (Brixton) [20] (set-shifting)

Participants were asked to predict the movements of a blue circle, which changes location after each response. Participants need to guess the concept behind these changes to make correct predictions. Occasionally, the pattern of movement changes and the participant has to abandon the old concept in favor of a new one. The total number of errors made on the test can be used to construct a scaled score. The greater the number of errors indicates the stronger the inefficiencies of set-shifting. The score yields between 1 and 10, with lower scores indicating the stronger the inefficiencies of set-shifting.

#### Wechsler intelligence scale for children (WISC)-IV

WISC-IV was measured during admission. One participant had been already been assessed using WISC-IV within 2 years, therefore this previous data was used.

#### Children’s version of eating attitude test with 26 items (chEAT-26)

The chEAT-26 is a self-report instrument of the characteristic psychopathology of AN [21]. The scale yields a total score between 0 and 78, with higher scores indicating worse eating attitude.

#### Autism-spectrum quotient (AQ)

The AQ [22] is a 50-question scale by participants’ parents. The scale yields a total score between 0 and 50, with higher scores indicating stronger autism trait.

The chEAT-26 and AQ were measured once before the CRT intervention.

#### Motivational ruler (MR)

MR was used to determine self-reported importance and ability to change [23]. It consists of two questions: (1) Importance to change.” How important is it for you to change and recover from your AN?” and (2) Ability to change.”How confident are you in your ability to change and recover from your AN?” Each rated 10-point Likert scale (1 = Not at all to 10 Very much).

#### Beck’s depression inventory (BDI)-II

The BDI-II yields a total score between 0 and 63, with higher scores indicating higher severity of depressive symptoms [24].

#### Rosenberg self-esteem scale (RSES)

The RSES is self-report instrument measuring an individual’s overall self-esteem [25]. The scale yields a total score between 10 and 50, with higher scores indicating higher self-esteem.

MR, BDI-II and RSES were assessed pre and post Group CRT.

#### Worksheet (each session)

The worksheets were documents of participants’ thinking about their thinking style, relation of the skills that they learnt through each session to real-life.

#### Satisfaction questionnaire (post-final session)

This questionnaire consisted of four open-ended questions: “What are the good things that you learned from CRT group?”; “How has the CRT group helped you?”; “What did you dislike about the CRT group?” and “What did you find difficult?”.

### Interventions

Participants received an usual treatment that including behavioral therapy, nutritional therapy, psychotherapy and psychoeducation group sessions. They also received four sessions of the CRT group. All sessions were provided weekly, and each session lasted for 40 min. Each session included tasks and reflective discussions of tasks and the session. The each session’s tasks were Session 1: Illusions task (switching how participants look at an image), Session 2: Geometric figures (describing a complex shape to a partner), Session 3: Switching Attention task (switching between two different pieces of information), and Session 4: a summary and occupational task (thinking about what skills the person doing an occupation would need). The therapist was a child psychiatrist (first author) and took part in the two-day CRT training workshop. Two child psychiatrists participated as facilitators in the first group. The second group consisted of a child psychiatrist and a nurse. The session program originating from the manual [9] and were adapted for younger adolescents by using more concrete demonstrations. Adolescents were given homework between sessions to practice what they learnt and to relate the skills real-life scenarios.

### Statistical analysis

Cohen’s d effect sizes were computed for the pre and post measures.

## Results

### Patient characteristics

Table 1 shows the patient characteristics.

**Table 1:** Participant demographic and clinical variables

Characteristics	Mean (SD)
Age (years)	13.86 (0.90)
Duration of illness (years)	1.06 (0.53)
BMI (kg/m^2^) (on admission)	12.89 (1.45)
Standard body weight ratio (%) (on admission)	64.00 (6.42)
WISC-IV	101.43 (15.67)
chEAT-26	21.57 (17.71)
AQ	16.14 (9.73)

*SD* standard deviation, *BMI* body mass index, *WISC* Wechsler intelligence scale for children, *chEAT-26* children’s version of eating attitude test with 26 items, *AQ* autism-spectrum quotient

Table 2 shows clinical variables, and neuropsychological and psychological assessment of the seven adolescents before and after the intervention. One participant dropped out, but she could carry out the remaining sessions on an individual basis, so seven adolescents were included in the final analysis. There were large effect sizes shown between the pre and post BMI and standard body weight ratio.

**Table 2:** Clinical variables, neuropsychological test scores, and psychological assessment between pre and post CRT

	Pre CRT mean (SD)	Post CRT mean (SD)	Effect size (d)
BMI (kg/m^2^)	14.77 (1.36)	16.01 (1.78)	0.80
Standard body weight ratio (%)	73.57 (7.14)	79.43 (8.61)	0.68
Neuropsychological measures			
ROCFT OI	2.41 (0.67)	2.33 (0.66)	0.13
ROCFT SI	1.36 (0.40)	1.45 (0.49)	0.22
ROCFT CCI	1.43 (0.38)	1.46 (0.44)	0.08
Brixton (the number of error)	19.6 (13.4)	17.8 (12.3)	0.15
Brixton (score)	5.3 (3.0)	6.0 (3.1)	0.25
Psychological measures (N = 6)			
BDI-II	19.2 (11.0)	14.2 (12.4)	0.47
RSES	30.7 (11.8)	28.8 (12.6)	0.17
MR-importance to change	7.0 (4.2)	8.3 (2.6)	0.45
MR-ability to change	7.3 (4.2)	8.3 (2.6)	0.31

One participant declined to complete the psychological measures (N = 6)

*CRT* cognitive remediation therapy, *SD* standard deviation, *BMI* body mass index, *ROCFT* the Rey–Osterrieth complex figure test, *OI* order index, *SI* style index, *CCI* central coherence index, *Brixton* the Brixton spatial anticipation test, *BDI-II* Beck’s depression inventory-II, *MR* motivational ruler, *RSES* Rosenberg self-esteem scale

### Neuropsychological and psychological measures

Dates were limited to provide reliable effect sizes due to small sample size.

### Feasibility and acceptability assessment of group CRT

Table 3 shows the outcomes for seven cases. Table 4 shows the participants’ reflecting about the relationship between their own thinking styles and real-life in Group CRT sessions. Table 5 shows feedback of Satisfaction Questionnaire. The dropout case was case 5 in the second group. She dropped out after two sessions because of high levels of anxiety and depression.

**Table 3:** Seven cases of clinical variables, neuropsychological test scores, and psychological assessment

Case	Sex	Age range	On admission			
			BMI (kg/m^2^)	Standard body weight ratio (%)	WISC	Duration of illness (years)
1	F	13–14	11.6	57	101	1
2	F	13–14	11.7	60	113	1.8
3	F	13–14	12.2	64	128	1.6
4	F	13–14	12.4	63	102	0.7
5	F	13–14	15.2	75	95	0.3
6	F	15–18	12.4	59	92	1.4
7	F	15–18	14.7	70	79	0.7

Case	Pre CRT												
	chEAT-26	AQ	BMI (kg/m^2^)	Standard body weight ratio (%)	MR		BDI	RSES	ROCFT			Brixton	
					Importance to change	Ability to change			OI	SI	CCI	The number of errors	Score
1	5	8	15.2	75	10	10	13	32	2.67	1.17	1.42	14	6
2	14	22	13.5	69					2.5	1.33	1.45	32	1
3	33	17	14.9	78	6	3	27	20	2	0.83	1.04	11	7
4	17	9	14.7	75	5	10	5	50	3.33	1.67	1.88	10	8
5	27	35	16.6	82	1	1	33	17	2.67	2	1.83	46	1
6	53	13	12.6	60	10	10	26	35	2.5	1.5	1.53	12	7
7	2	9	15.9	76	10	10	11	30	1.17	1	0.86	12	7

Case	Post CRT										
	BMI (kg/m^2^)	Standard body weight ratio (%)	MR		BDI	RSES	ROCFT			Brixton	
			Importance to change	Ability to change			OI	SI	CCI	The number of errors	Score
1	16.7	83	10	10	5	31	2	1.17	1.42	15	6
2	13.4	68					2	1.33	1.45	24	3
3	16.1	83	10	5	16	12	3	0.83	1.04	12	7
4	15.7	80	5	10	7	46	2.67	1.67	1.88	6	10
5	18.0	89	5	5	38	17	2.5	1.67	1.61	44	1
6	14.1	67	10	10	12	37	3	2	1.94	13	7
7	18.1	86	10	10	7	30	1.17	0.83	0.78	10	8

*CRT* cognitive remediation therapy, *WISC* Wechsler intelligence scale for children, *chEAT-26* children’s version of eating attitude test with 26 items, *AQ* autism-spectrum quotient, *BMI* body mass index, *MR* motivational ruler, *BDI-II* Beck’s depression inventory-II, *RSES* Rosenberg self-esteem scale, *ROCFT* the Rey–Osterrieth complex figure test, *OI* order index, *SI* style index, *CCI* central coherence index, *Brixton* the Brixton spatial anticipation test

**Table 4:** The participants’ examples of their reflecting about the relationship between their own thinking styles and real-life in Group CRT sessions

Session	The participants’ examples of their reflecting
1: Illusion task	
Case 1	“When I do something, firstly thinking is finishing, and secondly it is detail”
Case 2	“It is difficult to switch feelings in real-life”
Case 3	“In real-life I find it hard to see different perspectives of a situation, but I can do it with the guidance of a therapist and facilitators”
Case 4	“When I study for an exam, I tend to focus on one thing such as a weak point”
2: Geometric figures	
Case 1	“When I talk to someone, I start to talk without a plan. I can’t integrate information into the bigger picture”
Case 6	“I can’t do something in a variety of ways”
Case 7	“I tend to see details at first”
3: Switching Attention task	
Case 1 and 3	“When I get upset (ex. recital), it is difficult to switch feelings”
Case 6	“I like to make plans about a day (I dislike change)”
4: A summary and occupational task	
Case 1	“It is easy to switching feelings and behaviors when I accept a situation”
Case 3	“In real-life I tend to see in details. I tend to focus on one thing that I’m not good at”
Case 6	“I tend to focus on one thing. I want to be able to switch behaviors well”
Case 7	“I can switch feelings and behaviors in sports”

**Table 5:** Feedback of Satisfaction Questionnaire

Advantages of the CRT group (“What are the good things that you learned from CRT group?”)	
Case 1	“to be able to change more rapidly”
Case 3	“to know that different people have different opinions”
Case 6	“to think about new thinking styles and to change own thinking style in real-life”
Benefits of CRT skills to daily life (“How has the CRT group helped you?”)	
Case 1	“to facilitate both bigger picture thinking and detailed thinking styles”
Case 3	“to get many thinking styles”
Case 6	“to get another good point of view”
Disadvantages of the CRT group (“What did you dislike about the CRT group?” and “What did you find difficult?”)	
Case 3	“to make participants nervous because of in front of many facilitators”
Case 4	“homework”
Case 6	“difficult reflecting and generalize to real-life setting”

## Discussion

The objective of this study was to assess the feasibility and acceptability of a CRT group for younger adolescents with AN in a Japanese sample. The results of this study showed that a CRT group was feasible. First, there were large changes in adolescents’ weight. Second, there were medium changes in adolescents’ self-reported depressive symptoms and motivation (importance to change) to get better.

The results of this study also showed that a CRT group was acceptable for the adolescent involuntary inpatient. First, dropout rate (14%) was lower than previous reports (17, 33%) [11, 12] and dropout case could carry out CRT sessions on an individual basis. Secondly, adolescents’ feedback was positive. All participants were able to reflect on their own thinking styles, such as having difficulty with changing feelings and the tendency to focus on details in real-life. Case 1 reported that “I was able to change more easily, and adapt to bigger picture thinking styles and generalize to real-life settings”. Case 3 reported that “I had an awareness that different people have different opinions”. This may have resulted from opportunities to share the perspectives of other members in the group CRT. Adapting the CRT manual for younger adolescents was acceptable in a Japanese sample.

There could be two reasons to explain the small or negligible changes differences between the pre and post neuropsychological measures. First, the study was underpowered to detect statistical differences. Second, it could be difficult for some younger adolescents to generalize to real-life setting. Case 3 and 6 reflected and generalized to real-life setting well, did homework eagerly and her ROCFT score improved. Case 6 also took part in the sessions with her nurse. Homework is important for generalizing to real-life setting. Participating in homework was not possible for all the adolescents, and some of the adolescents’ negative feedback included finding homework difficult. In the future groups, homework could be more approachable for adolescents and future groups could include nurses to help facilitate and aid with generalizing to real-life settings.

Some participants reported that reflecting and generalizing to real-life settings was difficult. Therapists may need to devise effective means such as giving concrete examples and step by step instructions. Other negative feedback was that public speaking was too anxiety-provoking. Future groups could include more paired work to help the participants to feel more relaxed and comfortable.

One case dropped out because of high levels of anxiety and depression, but she could carry out on an individual basis. In such a case CRT on an individual basis could be more acceptable.

This was the first reported evaluation of a CRT group for younger adolescents in a Japanese sample who were more serious cases (mean BMI: 14.5) than the previous reports (mean BMI: 15.8) [11] that measured neuropsychological tests pre and post intervention. The participants were also younger (mean age: 13.9) than previous reports (mean age: 15.6 and 15.9) [6, 11].

This study design had four limitations. Firstly, it used an uncontrolled design and open trials, however the group CRT was carried out within the context of a real clinical setting, that indicates it is feasible to carry out the group in involuntary inpatients setting. Second, this study included a very small sample size, however, the results are preliminary and can be used to inform larger studies. Third, in this study no follow-up evaluation was set up, but two out of seven cases (29%) were re-hospitalized due to weight loss within one year in a post survey. Finally, all the participants were also given other therapies. It is difficult to attribute changes to group CRT. Future research could include a bigger sample size, follow-up evaluation and compare the CRT group with other types of therapies.

## Conclusions

Group CRT is likely to be feasible and acceptable for younger adolescent AN in a Japanese sample.

## Abbreviations

CRT: cognitive remediation therapy
AN: anorexia nervosa
TMCMC: Tokyo Metropolitan Children’s Medical Center
BMI: body mass index
ROCFT: the Rey–Osterrieth complex figure test
OI: order index
SI: style index
CCI: central coherence index
Brixton: the Brixton spatial anticipation test
WISC: Wechsler intelligence scale for children
chEAT-26: children’s version of eating attitude test with 26 items
AQ: autism-spectrum quotient
MR: motivational ruler
BDI: Beck’s depression inventory
RSES: Rosenberg self-esteem scale
SD: Standard deviation
